# Supplementary material for: 3D Printed Voltammetric Sensor Modified with an Fe(III)-Cluster for the Enzyme-Free Determination of Glucose in Sweat
Source: Biosensors (Basel). 2022 Dec 11;12(12):1156. doi: 10.3390/bios12121156 (PMC9775037; doi:10.3390/bios12121156)
Supplement: Supplementary file 1 [file biosensors-12-01156-s001.zip › biosensors-2002950-supplementary.pdf]

Supplementary Material

3D Printed Voltammetric Sensor Modified with an Fe(III)-Cluster for the Enzyme-Free Determination of Glucose in Sweat

Eleni Koukouviti [1], Alexios K. Plessas[2], Anastasios Economou[1], Nikolaos Thomaidis[1], Giannis S. Papaefstathiou[2], Christos Kokkinos[1]*

[1]Laboratory of Analytical Chemistry, Department of Chemistry, National and Kapodistrian University of Athens, 15771 Athens, Greece

[2]Laboratory of Inorganic Chemistry, Department of Chemistry, National and Kapodistrian University of Athens, 15771 Athens, Greece

TableS1. The reported crystal structures containing the $[Fe_3O(PhCOO_2)_6(H_2O)_3]^+$ moiety.

Refcode	Chemical Formula	Ref
DEQWEC	$[Fe_3O(PhCOO_2)_6(H_2O)_3]NO_3 \cdot 3MeCN$	[1]
IMUMUZ	$[Fe_3O(PhCOO_2)_6(H_2O)_3]ClO_4 \cdot 3MeCN$	[2]
TURJOF	$(C_5H_5ClN)[Fe_3O(PhCOO_2)_6(H_2O)_3](C_7H_7O_3S)_2 \cdot 2H_2O$	[3]

Table S2. Crystal data and structure refinement for [$Fe_3O(PhCO_2)_6(H_2O)_3$]·$PhCO_2$

**Empirical formula**	[$Fe_3O(PhCO_2)_6(H_2O)_3$]·$PhCO_2$
**Formula weight**	1085.39
**Temperature**	297 K
**Wavelength (Cu Ka)**	1.54059 Å
**Crystal system**	orthorhombic
**Space group**	P 2_1 2_1 2_1
	a = 18.52596 Å, α = 90.000°
**Unit cell dimensions**	b = 19.17324 Å, β = 90.000°
	c = 15.83827 Å, γ = 90.000°
**Volume**	5625.795 $Å^3$
**Z**	4
**Density (calculated)**	1.274 g/cm^3
**θ range for data collection**	3 to 30°
**Number of reflections (Bragg peaks)**	149
**Number of independent atoms**	105
**R_p**	3.883
**R_{wp}**	5.513
**Profile Function**	Pseudo-Voigt

Table S3. Atomic coordinates (x10[4]) and equivalent isotropic displacement parameters (Å[2]x10[3]) for [$Fe_3O(PhCO_2)_6(H_2O)_3$]·$PhCO_2$.

**Label**	**x**	**y**	**z**	**Ueq**
Fe(1)	3569	9262	5285	12.7
Fe(2)	3910	7857	6409	12.7
Fe(3)	2496	8831	6863	12.7
O(1)	3317	8648	6177	38
O(2)	2990	8686	7965	38
O(3)	3813	7882	7665	38
O(4)	3075	7182	6307	38
O(5)	2166	7824	6795	38
O(6)	4127	7614	5199	38
O(7)	3805	8528	4420	38
O(8)	4807	8445	6504	38
O(9)	4611	9342	5629	38
O(10)	1837	9023	5904	38
O(11)	2549	9264	4821	38
O(12)	2683	9852	7003	38
O(13)	3368	10143	5889	38
O(14)	3826	9936	4273	38
O(15)	4566	6998	6654	38
O(16)	1577	8954	7619	38
C(1)	4971	9019	6170	38
C(2)	5660	9357	6489	38
C(3)	5674	10071	6610	38
C(4)	6296	10376	6929	38
C(5)	6888	9984	7098	38
C(6)	6879	9280	6958	38
C(7)	6275	8966	6651	38
C(8)	4013	7908	4509	38
C(9)	4130	7470	3729	38

Label	x	y	z	Ueq
C(10)	4457	7756	3029	38
C(11)	4565	7321	2339	38
C(12)	4378	6662	2353	38
C(13)	4060	6371	3032	38
C(14)	3918	6783	3726	38
C(15)	2420	7280	6473	38
C(16)	1886	6701	6244	38
C(17)	1572	6703	5457	38
C(18)	1042	6174	5289	38
C(19)	927	5662	5904	38
C(20)	1200	5668	6663	38
C(21)	1695	6204	6853	38
C(22)	3510	8284	8156	38
C(23)	3751	8291	9073	38
C(24)	3743	7702	9531	38
C(25)	3995	7666	10380	38
C(26)	4240	8269	10716	38
C(27)	4256	8867	10284	38
C(28)	4007	8898	9443	38
C(29)	3033	10284	6558	38
C(30)	3037	11028	6862	38
C(31)	3484	11519	6529	38
C(32)	3499	12187	6857	38
C(33)	3027	12378	7443	38
C(34)	2565	11899	7796	38
C(35)	2558	11236	7481	38
C(36)	1934	9158	5132	38
C(37)	1312	9188	4554	38
C(38)	1043	8575	4182	38
C(39)	452	8620	3638	38
C(40)	153	9240	3474	38

Label	x	y	z	Ueq
C(41)	399	9840	3831	38
C(42)	986	9821	4373	38
O(17)	3747	12050	4213	38
O(18)	3982	12132	5600	38
C(43)	3727	12361	4912	38
C(44)	3367	13064	4947	38
C(45)	3784	13662	4963	38
C(46)	3476	14309	4961	38
C(47)	2738	14361	5004	38
C(48)	2328	13794	4976	38
C(49)	2638	13139	4992	38

Table S4. List of Bragg positions (shown as blue dashes in Figure 1) from the Rietveld Analysis of $[Fe_3O(PhCO_2)_6(H_2O)_3]\cdot PhCO_2$.

2θ	h k l	d	Fo	Fc	2θ	h k l	d	Fo	Fc
6.64	1 1 0	13.31	215.30	298.18	23.70	1 5 0	3.75	43.64	29.05
7.24	0 1 1	12.20	151.98	202.22	23.73	4 3 0	3.75	61.60	90.18
7.34	1 0 1	12.03	147.26	215.07	23.73	2 4 2	3.75	48.34	34.46
8.67	1 1 1	10.19	85.86	166.27	23.88	0 5 1	3.72	53.72	90.96
9.23	0 2 0	9.58	124.17	177.29	23.88	2 3 3	3.72	43.39	43.54
9.55	2 0 0	9.25	114.42	229.97	24.04	3 2 3	3.70	144.92	40.59
10.39	1 2 0	8.51	95.86	207.41	24.12	4 2 2	3.69	63.46	106.13
10.61	2 1 0	8.33	90.67	154.33	24.17	3 4 1	3.68	203.93	109.60
10.79	0 2 1	8.19	72.22	152.92	24.32	0 2 4	3.66	140.12	54.57
11.07	2 0 1	7.99	136.44	157.54	24.36	1 5 1	3.65	106.80	41.29
11.17	0 0 2	7.91	83.63	100.51	24.39	4 3 1	3.65	186.78	75.43

2θ	h k l	d	Fo	Fc	2θ	h k l	d	Fo	Fc
11.80	1 2 1	7.49	121.51	165.80	24.45	2 0 4	3.64	128.23	200.10
11.99	2 1 1	7.37	65.50	109.12	24.47	5 1 0	3.63	66.50	0.27
12.09	0 1 2	7.31	43.52	147.96	24.68	5 0 1	3.60	54.44	86.54
12.16	1 0 2	7.28	142.50	219.72	24.80	1 2 4	3.59	148.07	77.76
13.01	1 1 2	6.80	85.45	89.78	24.89	2 1 4	3.57	128.80	112.21
13.29	2 2 0	6.66	38.21	65.79	25.10	0 4 3	3.55	29.50	56.13
14.43	2 2 1	6.13	52.46	134.31	25.12	5 1 1	3.54	45.73	75.46
14.51	0 2 2	6.10	44.16	78.32	25.14	2 5 0	3.54	309.99	36.26
14.66	1 3 0	6.04	19.28	152.98	25.56	1 4 3	3.48	0.00	126.53
14.72	2 0 2	6.01	30.69	96.77	25.59	4 0 3	3.48	0.00	70.38
14.95	0 3 1	5.92	123.98	229.30	25.77	2 5 1	3.45	94.26	71.27
15.07	3 1 0	5.87	1.95	20.95	25.78	5 2 0	3.45	171.31	100.26
15.28	1 2 2	5.79	128.23	191.24	25.82	0 5 2	3.45	166.21	73.09
15.40	3 0 1	5.75	82.98	123.73	26.01	4 1 3	3.42	94.69	85.68
15.43	2 1 2	5.74	50.23	116.61	26.09	3 4 2	3.41	11.63	56.40
15.70	1 3 1	5.64	54.14	50.38	26.18	2 2 4	3.40	100.57	119.94
16.08	3 1 1	5.51	143.41	132.85	26.22	3 3 3	3.40	121.49	50.18
16.86	2 3 0	5.26	85.97	204.10	26.27	1 5 2	3.39	136.04	31.58
17.08	3 2 0	5.19	0.00	136.04	26.30	4 3 2	3.39	184.02	18.40
17.40	2 2 2	5.09	104.35	76.22	26.40	5 2 1	3.37	112.02	100.21
17.42	0 1 3	5.09	86.80	347.20	26.48	0 3 4	3.36	141.72	0.09
17.47	1 0 3	5.07	309.32	132.40	26.56	5 0 2	3.35	159.90	21.82
17.77	2 3 1	4.99	41.64	178.75	26.75	3 0 4	3.33	60.96	118.85
17.84	0 3 2	4.97	191.67	1.54	26.77	4 4 0	3.33	106.65	116.15
17.98	3 2 1	4.93	129.43	127.15	26.91	2 4 3	3.31	156.49	48.91
18.08	1 1 3	4.90	213.04	115.43	26.92	1 3 4	3.31	86.37	56.10
18.22	3 0 2	4.87	128.38	171.05	26.97	5 1 2	3.30	233.77	128.55

2θ	h k l	d	Fo	Fc	2θ	h k l	d	Fo	Fc
18.47	1 3 2	4.80	159.29	171.85	27.16	3 1 4	3.28	129.30	72.53
18.51	0 4 0	4.79	128.75	88.22	27.25	4 2 3	3.27	7.81	71.16
18.80	3 1 2	4.72	251.68	283.47	27.37	4 4 1	3.26	95.86	117.14
19.13	1 4 0	4.64	274.01	34.24	27.38	3 5 0	3.25	204.99	248.59
19.16	4 0 0	4.63	104.32	365.91	27.58	2 5 2	3.23	80.07	87.06
19.19	0 2 3	4.62	75.42	65.71	27.83	5 3 0	3.20	67.44	3.75
19.35	0 4 1	4.58	108.76	27.83	27.92	0 6 0	3.19	34.02	163.33
19.35	2 0 3	4.58	117.36	73.18	27.97	3 5 1	3.19	113.24	104.90
19.72	4 1 0	4.50	282.30	299.51	28.17	5 2 2	3.16	62.91	97.61
19.79	1 2 3	4.48	238.67	175.82	28.21	2 3 4	3.16	118.92	121.06
19.91	2 1 3	4.46	83.74	145.06	28.34	1 6 0	3.15	178.33	25.87
19.94	1 4 1	4.45	54.48	168.13	28.35	3 2 4	3.15	124.29	83.06
19.98	4 0 1	4.44	117.02	63.52	28.41	5 3 1	3.14	138.29	30.62
20.00	3 3 0	4.44	277.26	88.61	28.50	0 6 1	3.13	176.97	101.22
20.27	2 3 2	4.38	91.97	103.27	28.56	0 1 5	3.12	155.98	46.43
20.46	3 2 2	4.34	138.47	97.98	28.59	1 0 5	3.12	83.69	174.65
20.51	4 1 1	4.33	50.86	18.90	28.78	0 5 3	3.10	0.00	45.07
20.78	3 3 1	4.27	262.38	59.47	28.91	1 6 1	3.09	0.00	15.33
20.87	2 4 0	4.25	103.73	128.34	28.92	6 0 0	3.08	0.00	160.83
21.31	4 2 0	4.17	65.67	141.11	28.98	1 1 5	3.08	41.17	99.11
21.48	2 2 3	4.13	190.50	172.51	29.02	3 4 3	3.07	46.84	63.06
21.62	2 4 1	4.11	43.36	163.10	29.09	4 4 2	3.07	54.65	24.91
21.67	0 4 2	4.10	177.83	1.45	29.19	1 5 3	3.06	56.21	51.34
21.84	0 3 3	4.07	102.45	83.61	29.21	4 3 3	3.05	58.30	139.95
22.04	4 2 1	4.03	93.10	143.54	29.26	0 4 4	3.05	50.93	72.00
22.15	3 0 3	4.01	189.04	83.03	29.30	6 1 0	3.05	32.60	62.69
22.21	1 4 2	4.00	132.92	50.77	29.45	5 0 3	3.03	34.21	23.41

2θ	h k l	d	Fo	Fc	2θ	h k l	d	Fo	Fc
22.24	4 0 2	3.99	218.49	328.49	29.48	6 0 1	3.03	30.41	152.10
22.37	1 3 3	3.97	128.33	42.87	29.57	2 6 0	3.02	77.48	53.51
22.46	0 0 4	3.96	0.00	43.23	29.66	3 5 2	3.01	48.76	61.46
22.64	3 1 3	3.92	0.00	99.69	29.66	1 4 4	3.01	49.21	37.87
22.72	4 1 2	3.91	1.06	81.49	29.69	4 0 4	3.01	87.26	4.19
22.94	0 1 4	3.87	72.58	155.95	29.71	0 2 5	3.01	106.29	49.23
22.96	3 3 2	3.87	81.18	148.15	29.81	2 0 5	2.99	106.82	5.65
22.97	1 0 4	3.87	228.59	92.67	29.83	5 1 3	2.99	45.87	31.86
23.44	1 1 4	3.79	30.59	3.02	29.85	6 1 1	2.99	44.40	14.26
23.50	3 4 0	3.78	69.95	24.63					

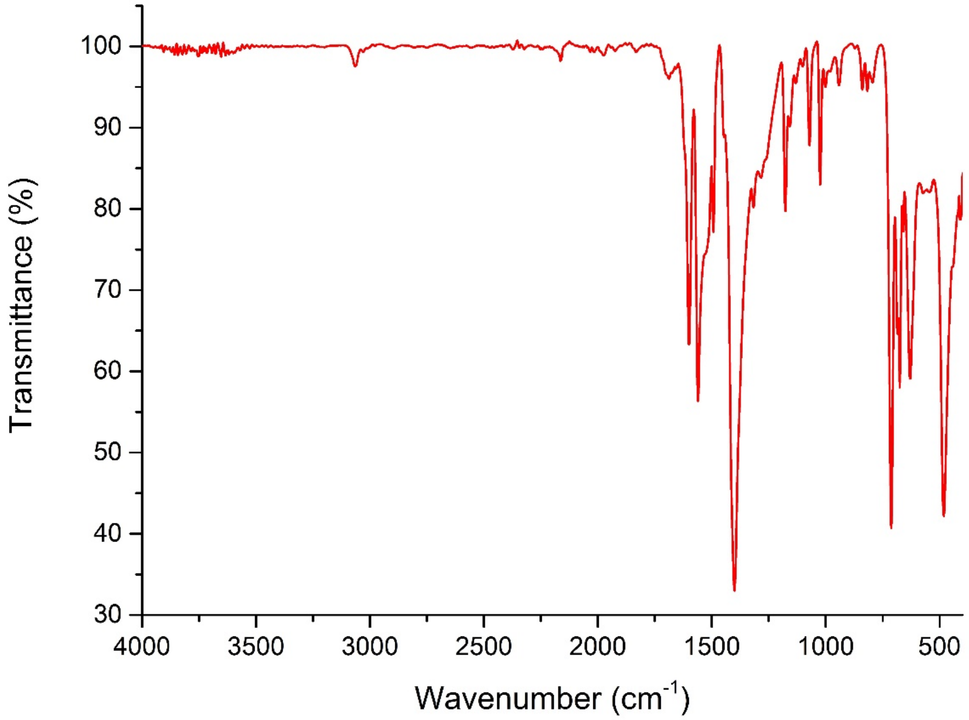

Figure S1. The IR spectrum (ATR) of $[Fe_3O(PhCO_2)_6(H_2O)_3]{\cdot}PhCO_2$ in the 450 – 4000 cm^{-1} range.

Particle size analysis

The particle size was calculated using ImageJ v. 1.53t software [4], using an image taken on a Leica M205 C stereoscope equipped with a Leica DMC5400 - 20 Megapixel camera. The image was taken from a sample of $[Fe_3O(PhCO_2)_6(H_2O)_3]{\cdot}PhCO_2$, dispersed in water, on a glass slide.

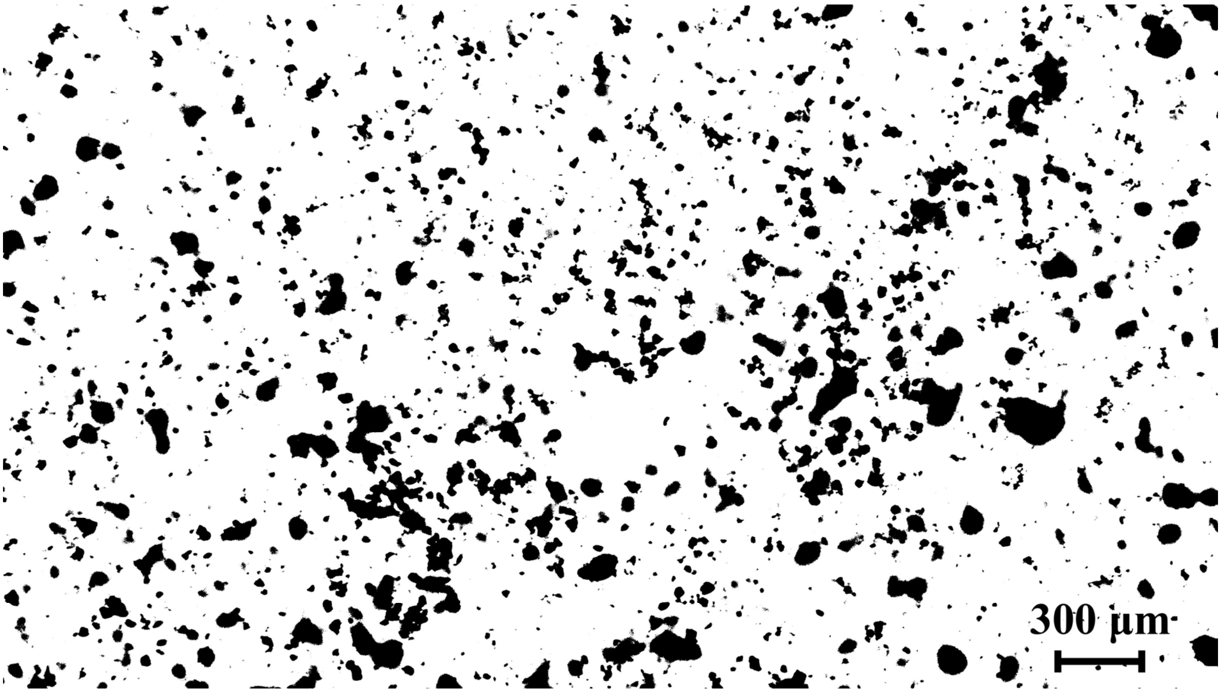

Figure S2. Image of $[Fe_3O(PhCO_2)_6(H_2O)_3]{\cdot}PhCO_2$ *dispersed in water on a glass slide.*

From the gaussian fit of the measurements from ImageJ, the particle size ranges from 50 to 230 µm and the average particle size is 54.66 µm.

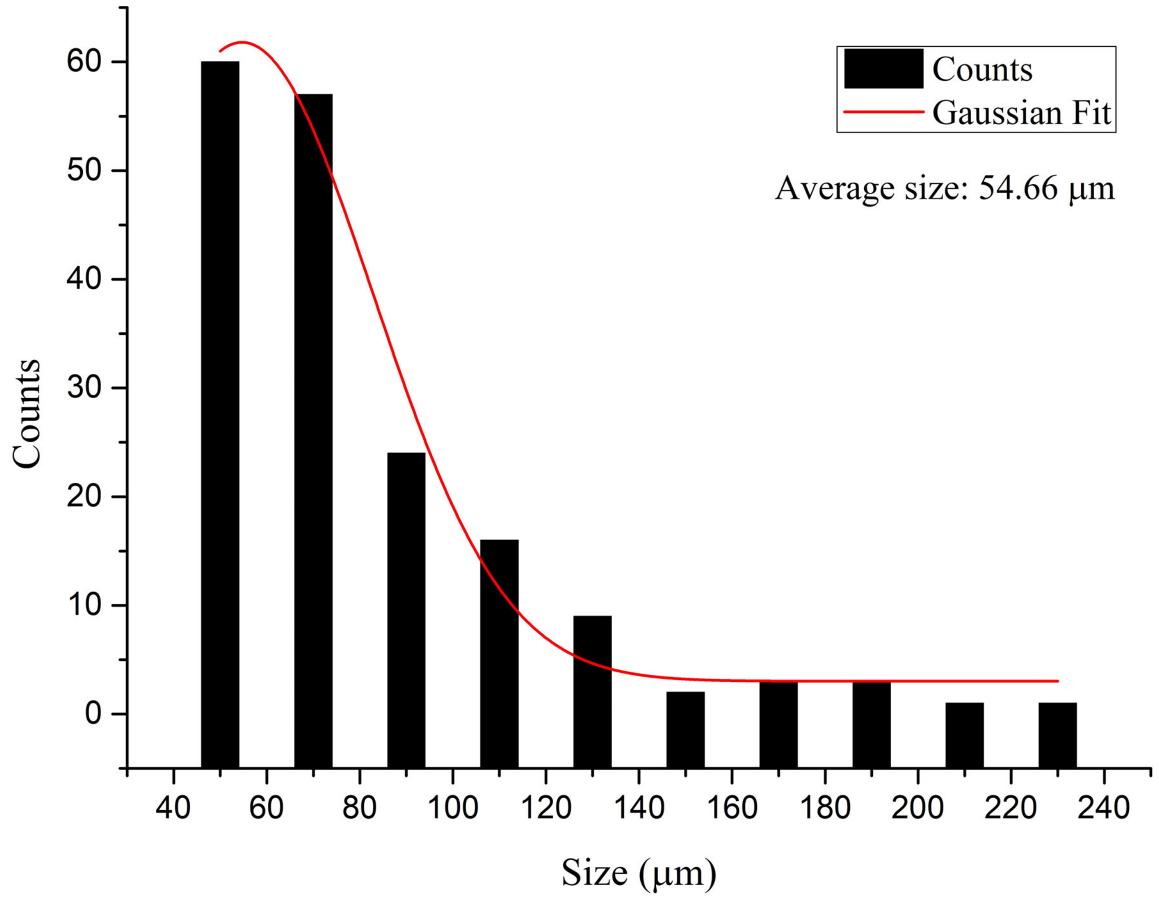

Figure S3. Histogram of particle sizes ranging between 50 and 230 μm (black bars), and gaussian fit (red line).

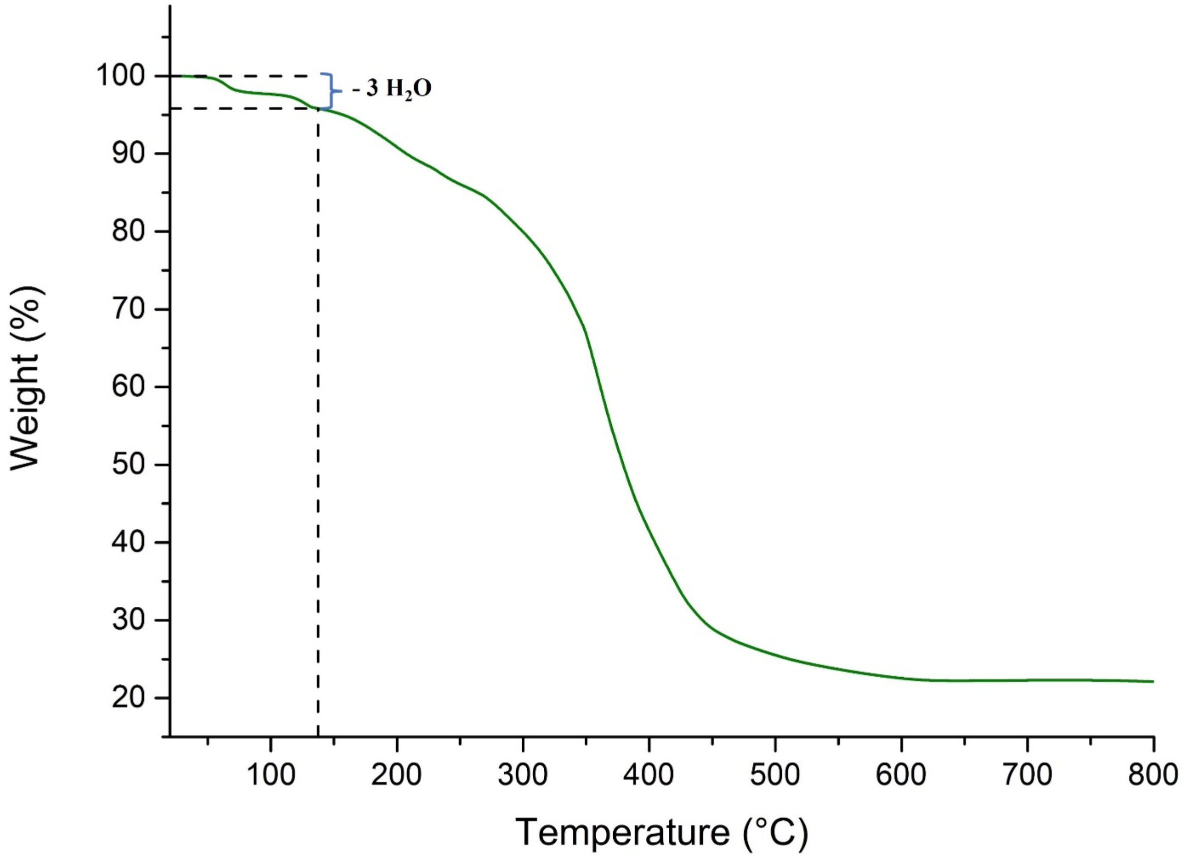

Figure S4. Thermogravimetric analysis (TGA) graph of [$Fe_3O(PhCO_2)_6(H_2O)_3$]·$PhCO_2$ in the 25 – 800 ^oC temperature range.

References

[1] Psycharis, V., Raptopoulou, C. P., Boudalis, A. K., Sanakis, Y., Fardis, M., Diamantopoulos, G. & Papavassiliou, G. Syntheses, Structural, and Physical Studies of Basic Cr III and Fe III Benzilates and Benzoates: Evidence of Antisymmetric Exchange and Distributions of Isotropic and Antisymmetric Exchange Parameters. *Eur. J. Inorg. Chem.* **2006**, 2006, 3710–3723. https://doi.org/10.1002/ejic.200600306.

[2] Georgopoulou, A. N., Sanakis, Y., Psycharis, V., Raptopoulou, C. P. & Boudalis, A. K. Mössbauer spectra of two extended series of basic iron(III) carboxylates [Fe3O(O2CR)6(H2O)6]A (A− = ClO4 −, NO3 −). *Hyperfine Interact.* **2010**, 198, 229–241. https://doi.org/10.1007/s10751-010-0179-2.

[3] Cadelnic, I., Shova, S., Simonov, Y.A., Cendrowska, E., Gdaniec, M., Jovmir, T., Turta, C. and Bulhac, I. Crystal and molecular structure of [Fe3O(C6H5COO)6 (H2O)3]·3-Cl-PyH·(CH3C6H4SO3)2·2H2O. *Pol. J. Chem.* **1997**, 71, 501–508.

[4] Schneider, C. A., Rasband, W. S. & Eliceiri, K. W. NIH Image to ImageJ: 25 years of image analysis. Nat. Methods **2012**, 9, 671–675.
